# Equity in Health Policy for Persons With Disabilities in Brazil: Spatial Distribution of Specialised Rehabilitation Centres

**DOI:** 10.1002/hpm.70010

**Published:** 2025-07-18

**Authors:** Paulo Henrique dos Santos Mota, Bianca Tomi Rocha Suda, Patricia Marques Moralejo Bermudi, Francisco Chiaravalloti Neto, Aylene Bousquat

**Affiliations:** ^1^ Department of Policies Management and Health School of Public Health University of São Paulo São Paulo Brazil; ^2^ Postgraduate Program in Public Health School of Public Health University of São Paulo São Paulo Brazil; ^3^ Department of Epidemiology School of Public Health University of São Paulo São Paulo Brazil

**Keywords:** health planning, health system, persons with disabilities, rehabilitation services, spatial analysis

## Abstract

**Objective:**

To analyse the spatial distribution of Specialised Rehabilitation Centres (CERs) in Brazil, considering the prevalence of persons with disabilities (PWD), socioeconomic factors, and health financing.

**Methods:**

An ecological study design was employed, using descriptive and Bayesian spatial regression analyses on data from 438 health regions in Brazil. The presence or absence of CERs in these regions was the main outcome. Covariates included PWD population, socioeconomic indicators, health service funding, and health system factors.

**Results:**

The study revealed that CERs are present in only 32% of health regions, with significant associations between CER implementation and factors such as monthly per capita household income, health expenditure per inhabitant, and regional GDP. Notably, the increase in PWD numbers did not directly correlate with CER implementation at the regional level.

**Conclusion:**

The implementation of CERs is influenced by economic and health service factors, not just by the prevalence of PWD. To improve equity in access, it is essential to prioritise CER implementation in regions with higher rehabilitation needs and better utilise available data on disability demographics. Comprehensive, integrated care for PWD requires interdisciplinary and intersectoral actions.

## Summary

- *Low coverage of CERs in Brazil:* Only 34.9% of health regions have Specialised Rehabilitation Centres (CERs), with significant disparities in their distribution across the country.
- *Economic factors impact CER implementation:* The study identified that CER implementation is positively associated with an increase in monthly per capita income, public health spending per inhabitant, and the GDP of the health region.
- *Disability prevalence is not sufficient to justify implementation:* The higher concentration of persons with disabilities (PWD) in certain regions does not directly translate into greater CER implementation, highlighting the need to consider other factors for prioritisation.
- *Regional inequality in rehabilitation needs:* The Northeast region has the highest concentration of rehabilitation needs, yet the availability of CERs remains insufficient, perpetuating disparities in access to health services for PWD.
- *Importance of qualified data for public policies:* The study emphasizes the need for updated and accurate data on the PWD population to guide the implementation of more equitable and effective health policies.

## 1 Introduction

Current estimates indicate that approximately 16% of the global population lives with some form of disability [1]. Persons with disabilities (PWD) face greater difficulties in accessing primary health care services, medical specialities, hospitals, or rehabilitation [2]; moreover, they are not a homogeneous group and their health needs vary. Some necessitate complex, intensive, and specialised treatments, typically provided in facilities with advanced technology [3]. However, the majority require rehabilitation services, which can be offered in primary health care settings or in medium complexity services [4].

The demand for rehabilitation services is increasing due to three major factors. Firstly, the global population is ageing. Secondly, the prevalence of noncommunicable diseases is rising. Thirdly, the world has recently experienced significant upheaval due to the global pandemic of the coronavirus (SARS‐CoV‐2). On a global scale, the demand for rehabilitation services has grown considerably, with 2.41 billion individuals requiring such services in 2019, marking a 63% increase since 1990 [1, 5]. Despite this growing demand, rehabilitation services remain under‐resourced and undervalued, particularly in low‐income countries where the growth in rehabilitation needs is most pronounced [6].

The demand for rehabilitation services has been rising globally, with certain regions experiencing particularly significant increases. In the Asia‐Pacific and Latin America regions, over two‐thirds of non‐fatal health loss is attributable to conditions that could be addressed by rehabilitation, underscoring the pressing need for these services [7]. The Western Pacific region is particularly noteworthy in this regard, with an estimated 610 million individuals in need of rehabilitation services [1]. A similar trend is observed in the BRICS nations, where the demand for rehabilitation has grown substantially, particularly due to musculoskeletal and pain conditions [8].

According to data from the 2010 Brazilian demographic census, 6.7% of the country's population has some form of disability. When analysed according to specific impairment types, the data reveals that 3.4% of Brazilians are visually impaired, 2.3% are physically impaired, 1.4% are intellectually impaired, and 1.1% are hearing impaired. A 2022 study based on functional difficulties found that 3.4% of Brazilians have difficulty walking or climbing steps, 3.1% have difficulty seeing, even when wearing glasses or contact lenses, 2.6% have difficulty learning, remembering things or concentrating, and 1.2% have difficulty hearing, even when wearing hearing aids [9].

Implementing policies, actions, and services aimed at this population is part of *the third* Sustainable Development Goal of the United Nations *(UN)*. It encourages *countries* to guarantee equitable access to quality and affordable health services [10].

At the beginning of the 1990s, Brazil implemented its Unified Public Health System (SUS, *Sistema Único de Saúde*), whose guidelines established universal access, equity, and comprehensive care [11]. As *SUS began to respond* to the health demands of *PWDs*, public rehabilitation services were implemented, especially in state capitals, urban areas, and places with greater socioeconomic development.

The National Health Policy for Persons with Disabilities, established in 2002, signified a pivotal moment in the incorporation of this demographic into the Brazilian Unified Health System (SUS). The policy delineated comprehensive guidelines for health promotion, prevention, treatment, and rehabilitation. The overarching objective of the policy was to ensure comprehensive healthcare, with a particular focus on providing access to specialised services, rehabilitation, and assistive technologies. In 2011, the National Plan for the Rights of Persons with Disabilities—Living without Limits (*Plano Nacional dos Direitos da Pessoa com Deficiência—Vivre sem Limites*) was established with the participation of fifteen ministries and the National Council for Persons with Disabilities. The ‘Living without Limits’ policy proposed the articulation of government policies in the areas of education, social inclusion, accessibility and health [12]. In 2012, the Ministry of Health established the Care Network for Persons with Disabilities (*RCPD, Rede de Cuidados à Pessoa com Deficiência*), breaking with the previous model and aiming to offer comprehensive care through an articulated and interconnected health care network (HCN) involving primary health care (PHC) services, speciality outpatient clinics, rehabilitation centres, hospital care, urgency and emergency services, in addition to orthopaedic workshops and dental centres.

Ideally, the RCPD should be planned based on epidemiological criteria, supported by multidisciplinary teams, with care regulation defined by priority criteria, guided by continuous care flows between network services [13] and with coverage within the Health Regions, defined as ‘Continuous geographic space, consisting of a grouping of neighbouring municipalities, defined based on cultural, economic and social identities and shared communication networks and transport infrastructure, aiming at integrating the organization, planning and implementation of health actions and services’ [14]. These are guided by population size, existing health resources and equipment, the forms of access and, to a lesser extent, the spatial size of the territory. They aim to optimise the management of the system, rationalise resources and institutional support [15, 16].

This is a strategy adopted by the Brazilian federal government to integrate the network of health services and existing rehabilitation facilities, as well as fund the implementation and costs of specialised rehabilitation centres *(CER, Centro Especializado em Reabilitação)*, aiming to reduce inequalities and increase access. The CERs are rehabilitation services organised by type of disability (hearing, intellectual, physical and visual) and constitute a core element in the Brazilian response to the health care of PWD.

The principal rehabilitation service financed by the Brazilian Ministry of Health is the *Centro Especializado em Reabilitação* (CER, Specialised Rehabilitation Centre). These centres are organised according to specific types of disabilities—namely hearing, intellectual, physical, and visual—and constitute a central component of the national healthcare strategy for persons with disabilities (PWDs). A single CER may offer healthcare services for more than one type of disability.

CERs are designed to provide diagnostic, therapeutic, and rehabilitative services, in addition to the provision, adaptation, and maintenance of assistive technologies. The care model is interdisciplinary, involving coordinated actions among health professionals, caregivers, and family members. Interventions are guided by the individualised needs of each user, taking into account the extent to which the disability affects functional capacity, as well as associated clinical, emotional, environmental, and social determinants. This approach reflects a biopsychosocial understanding of disability and aligns with international guidelines on comprehensive and person‐centred care.

Given that the CER constitutes a priority component of healthcare provision for PWDs, it is essential that these services be allocated to areas with higher concentrations of potential beneficiaries. This strategy is fundamental to reducing health inequalities and improving service equity.

Accordingly, this study aims to analyse the spatial distribution patterns of CERs across Brazilian territory, considering the prevalence of PWDs in the population, socioeconomic indicators, and healthcare financing structures.

### 1.1 Method

An ecological study design was employed, incorporating both descriptive and Bayesian spatial regression analyses, with the 438 Health Regions of Brazil serving as the units of analysis. The primary outcome was defined as the ‘presence or absence of specific CERs in the Health Regions.’

The explanatory variables were organised into four groups of indicators. The first group captured the number and percentage of persons with disabilities (PWD) in the population, based on data from the most recent national census conducted in 2010 [17], with updates made in 2018 [9] to align with the Washington Group recommendations [18].

The third group is related to the funding of health services; therefore, the following variables were used: Total value of financial resources for health transferred from the federal government to the municipalities and; Total public health expenditure per inhabitant. The last group comprises conditioning factors of the health system. In this case, the chosen covariates were: the percentage of the population covered by Primary Health Care and the typology of supply and complexity of health services as proposed by Viana et al. (2015) [16]. The covariates and the source of data collection are shown in Table 1.

**TABLE 1:** Covariates used in the exploratory analysis stage.

List of main outcomes	Source
Presence or absence of CER—Hearing disability	Ministry of Health: General Health coordination for persons with disabilities (CGSPD/DAET/SAES/MS)
Presence or absence of CER—Intellectual disability	
Presence or absence of CER—Physical disability	
Presence or absence of CER—Visual disability	
Covariates considered in exploratory analyses	
Number of persons with the specific disability(ies)	IBGE: Census 2010—Data updated in 2018
% Of persons with the specific disability(ies)	
Total GDP of the health region 2010	Brazilian institute of geography and statistics—IBGE: National accounts system
Monthly per capita household income	IBGE: Census 2010
Percentage of the population benefiting from the	CAIXA: Payroll of the *Bolsa Família* programme; IBGE: Population projections
*Bolsa família* programme 2014	
Health costs per inhabitant	DATASUS: SIOPS; IBGE: Population projections
SUS Transfer resource per inhabitant	
Supply and complexity of health services	Region and networks research [16]
Primary Health care coverage	DATASUS

Abbreviation: DATASUS, department of informatics of the brazilian unified health system.

*Source:* The authors.

The second group encompassed socioeconomic indicators, recognising that PWDs are disproportionately affected by poverty and income inequality [19]. The following indicators were included: the Gross Domestic Product (GDP) of each Health Region, per capita household income, and the percentage of the population receiving benefits from the federal cash transfer programme, *Bolsa Família* [20].

The third group addressed healthcare financing. Variables included: the total financial resources for health transferred from the federal government to municipalities, and total public health expenditure per capita.

The fourth group comprised health system determinants. This included the percentage of the population covered by Primary Health Care (PHC), and the typology of healthcare supply and service complexity, as proposed by Viana et al. (2015) [16]. These covariates, along with their respective data sources, are presented in Table 1.

Initially, exploratory analyses were conducted to examine associations between covariates and the presence or absence of specific CERs across Health Regions. Separate analyses were conducted for each outcome, corresponding to distinct models. The distribution of each covariate was assessed to detect outliers or anomalous values, and graphical methods were employed to explore patterns and relationships with the dependent variable. To assess multicollinearity, the Variance Inflation Factor (VIF) was calculated for all covariates within each model; variables with VIF values greater than 3 were excluded to improve model stability. All analyses were conducted using R packages, following the methodological recommendations of Zuur et al. (2010) [21].

The modelling was performed using a binomial distribution, as the outcome variable is dichotomous, representing the presence or absence of the event of interest. To account for the spatial dependence among the Health Regions, spatial random effects were incorporated following the approach proposed by Besag et al. (1991) [22]. This approach models spatial correlation by including random effects that capture the influence of neighbouring regions, assuming that the outcome in one region may be correlated with the outcomes in adjacent areas. This is crucial for properly modelling spatial data, as ignoring spatial dependencies could lead to biased or inefficient estimates.

To improve the interpretability of the parameters and enhance the model's computational stability, we employed the BYM2 reformulation proposed by Riebler et al. (2016) [23]. In this approach, the model's two spatial random effects, the structured component (which captures spatial correlation) and the unstructured component (which accounts for independent, local variation), are reparameterised into a single spatial effect. This is achieved by introducing a mixing parameter that controls the balance between the spatial structure (the structured component) and the random noise (the unstructured component). This reparameterisation improves both the numerical stability of the model and the interpretability of the parameters, as it reduces potential issues with parameter identifiability, which can be common in models with multiple random effects.

Latent Gaussian Bayesian models were used, with binomial probability distribution, run for each of the outcomes, considering the set of selected covariates, as a result of the collinearity verification step [24]. Continuous variables were standardized by subtracting the mean and dividing by the standard deviation. This standardisation ensures that all variables are on the same scale, improving comparability. It also facilitates the interpretation of model coefficients and enhances the numerical stability and robustness of the model. As for the categorical variable, the Socioeconomic Group, it was recoded into three categories and transformed into a dummy variable [21].

To select the best model, we applied the Backward regression method [25], which iteratively removes one variable at a time while assessing the Deviation Information Criterion (DIC). This process continues until the model with the lowest DIC value is identified, indicating the best fit.

For all models, neighbourhood relationships between Health Regions were defined using a Queen‐type contiguity matrix, where a region is considered a neighbour if it shares either a boundary or a vertex with another region. This approach was preferred over the Rook‐type contiguity matrix, which considers only shared boundaries, as it provides a more comprehensive definition of spatial relationships. This is particularly relevant when Health Regions have irregular shapes. By incorporating both edges and corners, the Queen criterion ensures that spatial dependence is adequately captured, minimising the risk of excluding relevant neighbouring influences. The modelling was performed in a Bayesian context using the integrated nested Laplace approximation (INLA) approach [26]. Non‐informative priors were considered for fixed effects and penalised complexity priors for random effects [27]. The programme R version 4.0.5 and the packages INLA [28] and INLAOutPus [29] were used to perform the analyses and to present the results. The maps were built using QGIS3.12. As this study employed solely secondary data, it was not subject to the requirement of ethical approval.

## 2 Results

At the time of data collection, Brazil had a total of 153 (34.9%) Health Regions with a CER. This coverage corresponds to a territorial area encompassing approximately 104 million inhabitants, indicating that 44.3% of the Brazilian population lives in regions where this essential service is entirely absent (Table 2).

**TABLE 2:** Distribution of health regions with specialised rehabilitation centres (CER) according to the types of disabilities covered.

	Health regions with CER	
	n	%
Type of disability		
Hearing	69	15.8
Intellectual	132	30.1
Physical	147	33.6
Visual	29	6.6
Total	153	34.9
Disabilities covered by the CER		
H‐I‐P‐V	28	6.4
H‐I‐P	18	4.1
H‐I‐V	0	0.0
H‐P‐V	7	1.6
I‐P‐V	10	2.3
H‐I	4	0.9
H‐P	12	2.7
H‐V	0	0.0
I‐P	70	16.0
I‐V	2	0.5
P‐V	2	0.5
Total	153	34.9

Abbreviations: H, hearing; I, intellectual; P, physical; V, visual.

*Source:* The authors.

**FIGURE 1:**
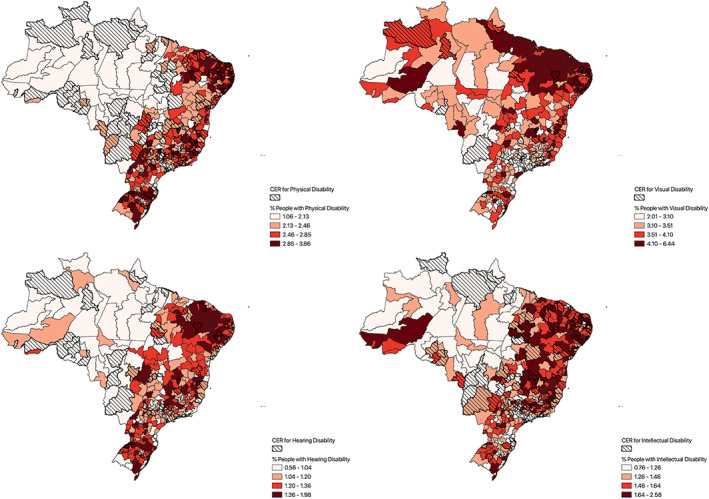
Distribution of CER and percentage of persons with disabilities (Physical, Visual, Hearing and Intellectual). Health Regions, Brazil. *Source:* The authors.

When disaggregated by type, there were 143 Health regions with a CERs for physical disabilities, 132 for intellectual disabilities, 69 for hearing disabilities, and 29 for visual disabilities. It is noteworthy that all CERs receive public funding from the Brazilian government. Figure 1 presents the spatial distribution of CERs alongside the distribution of PWDs across Brazil.

Regarding the distribution of PWDs, the Northeast region concentrates a substantial proportion of Health Regions within the upper quartile (75th percentile) for disability prevalence: 53.1% for intellectual disabilities, 44.1% for physical disabilities, 72.5% for visual disabilities, and 51.4% for hearing disabilities. In contrast, Health Regions falling below the 25th percentile are predominantly located in the Southeast—accounting for 33.6% of low‐prevalence regions for intellectual disabilities, 56.3% for visual disabilities, and 32.4% for hearing disabilities—and in the North, which includes 36.4% of the lowest prevalence regions for physical disabilities.

Despite these disparities in need, CERs are predominantly located in the Northeast and Southeast regions, with each region hosting approximately one‐third of the total CERs available in the country.

Table 3 presents the results of the final statistical models, highlighting the variables that remained significant, along with their corresponding odds ratios (OR) and 95% confidence intervals (95% CI).

**TABLE 3:** Posterior means of the odds ratios (OR) and 95% confidence interval (CI) for the covariates of the spatial models, regarding the presence of specific CERs in the Health Region, according to the types of hearing, intellectual, physical, visual disabilities and the total of the four types. health regions, Brazil.

Models	OR	CI (95%)
Hearing disability		
Interspect	0.1	(0.1–0.1)
Number of persons with hearing disability	2.0	(1.2—3.2)
Monthly per capita household income	2.2	(1.3–3.9)
SUS transfer of resources per inhabitant	1.6	(1.1–2.3)
Health costs per inhabitant	0.5	(0.3–0.9)
Intellectual disability		
Interspect	0.4	(0.3–0.5)
Number of persons with intellectual disabilities	3.6	(2.2. ‐ 5.8)
SUS Transfer of resources per inhabitant	1.5	(1.2–1.9)
Physical disability		
Interspect	0.5	(0.4–0.6)
SUS Transfer of resources per inhabitant	1.4	(1.1–1.7)
Monthly per capita household income	1.9	(1.2–3.1)
Number of persons with physical disabilities	3.4	(1.8–6.1)
Visual disability		
Interspect	0.1	(0.0–0.1)
Number of persons with visual disability	2.4	(1.6—3.6)
Total of the four types		
Interspect	0.5	(0.4–0.6)
Monthly per capita household income	2.3	(1.4–3.6)
SUS Transfer of resources per inhabitant	1.4	(1.1–1.8)
Total GDP of the Health region	3.0	(1.6—5.7)

*Source:* The authors.

In the model encompassing all four types of disabilities, positive and statistically significant associations were observed for the covariates *Monthly* per capita *household income*, *SUS transfer of resources per inhabitant*, and *Total GDP of the Health Region (2020)*. Specifically, the likelihood of a Health Region having a CER that offers services for any of the four disability types increases by 130% with a one standard deviation increase in *Monthly* per capita *household income*; by 40% with a one standard deviation increase in *SUS transfer of resources per inhabitant*; and by 200% with a one standard deviation increase in *Total GDP of the Health Region*.

In the model for hearing disability, the covariate *Health expenditure per inhabitant* was inversely associated with CER presence, whereas *Monthly* per capita *household income*, *Number of persons with hearing disability*, and *SUS transfer of resources per inhabitant* showed positive and significant associations. A one standard deviation increase in the *Number of persons with hearing disability* was associated with a 100% increase in the odds of having a CER for this condition. Similarly, a one standard deviation increases in *Monthly* per capita *household income* and *SUS transfer of resources per inhabitant* increased the odds by 120% and 60%, respectively. In contrast, a one standard deviation increase in *Health expenditure per inhabitant* was associated with a 50% reduction in the likelihood of a Health Region having a CER for hearing disabilities.

For intellectual disability, the variables that remained statistically significant were *SUS transfer of resources per inhabitant* and *Number of persons with intellectual disabilities*. An increase of one standard deviation in the former was associated with a 40% increase in the odds of having a CER for intellectual disability, while a similar increase in the number of persons with this disability resulted in a 260% increase in the odds.

In the physical disability model, a one standard deviation increases in *SUS transfer of resources per inhabitant* raised the odds of CER presence by 40%, and a similar increase in *Monthly* per capita *household income* raised the odds by 90%. Moreover, an increase of one standard deviation in the *Number of persons with physical disabilities* was associated with a 240% increase in the likelihood of a Health Region hosting a CER for this type of disability.

Finally, in the model for visual disability, the only covariate that remained statistically significant was the *Number of persons with visual disability*. A one standard deviation increase in this variable was associated with a 140% increase in the odds of a Health Region having a CER specialised in visual disabilities.

## 3 Discussion

One of the fundamental prerequisites for the effective implementation of public health policies is the accurate identification of population needs, guided by epidemiological evidence. At the same time, the feasibility of these policies is contingent upon the availability of financial resources. The findings of this study suggest that both factors are critical in explaining the implementation patterns of *Centros Especializados em Reabilitação* (CERs) across Brazil. Specifically, the results indicate that CER implementation is influenced by economic indicators, increased investment in health, and population needs—indirectly reflected by the number of persons with disabilities (PWD) in each Health Region.

Despite their importance, limited data are available regarding the quantity, distribution, and types of disability in Brazil. The last national census was conducted in 2010, with a new edition originally scheduled for 2020 but postponed to 2022 due to the COVID‐19 pandemic. The census data presented in this study show a higher prevalence of PWDs in the Northeast region, underscoring the need for regionally tailored health planning and service allocation.

It is important to account for the temporal *gap* in data on the population of persons with disabilities (PWD) when interpreting the findings presented in this study. Between the last national census in 2010 and the most recent one in 2022, Brazil experienced significant public health events, notably the COVID‐19 pandemic and the Zika virus epidemic [30]. The latter was associated with a sharp rise in the number of children born with neuropsychomotor development delays and microcephaly. Between 2000 and 2014, the country recorded an annual average of 164 cases of microcephaly, whereas in 2015 alone, 1608 cases were reported [31].

Furthermore, the period between 2000 and 2013 saw a substantial increase in traffic accidents. During this time, there were 1,747,191 hospitalizations attributed to such incidents, with 23.5% of those cases presenting diagnoses suggestive of physical sequelae [32]. These epidemiological shifts likely contributed to changes in the demand for rehabilitation services, underscoring the need for up‐to‐date, continuous data to inform the planning and allocation of resources for PWD.

In addition to generating immediate and acute healthcare needs, the COVID‐19 pandemic has also created new and persistent demands that must be addressed by health systems. Among individuals who survive the acute phase of the disease, medium‐to long‐term symptoms—collectively referred to as ‘Long COVID’—have been documented. These include functional impairments such as fatigue, dyspnoea, arthralgia, sleep disturbances, and chest pain. The sequelae associated with Long COVID encompass multiple systems, including dermatological, respiratory, cardiovascular, musculoskeletal, neurological, renal, and mental health domains [33, 34, 35].

Healthcare for PWD must be delivered through a comprehensive range of actions and services encompassing health promotion, disease prevention, treatment, and rehabilitation. Although federal regulations concerning the RCPD formally encompass this continuum of care, financial resources are predominantly allocated to the implementation of CERs and Orthopaedic Workshops [12]. Studies has demonstrated that the Brazilian federal government and the Ministry of Health hold significant power to influence policy through financial incentives, despite often maintaining a passive, normative, and low‐accountability role in the direct provision of services [36]. Over the past 3 decades, financial transfers have increased, particularly through incentives aimed at supporting the implementation of health actions and services [37]. However, within the scope of the RCPD, funding remains primarily focused on rehabilitation services, with no dedicated financial mechanisms to support the development or integration of services into a comprehensive and coordinated health care network.

Economic factors appear to be associated with the implementation of CERs, particularly indicators such as the transfer of resources from the Unified Health System (SUS) by the federal government to municipalities on a per capita basis, monthly per capita household income, and the Gross Domestic Product (GDP) of the Health Region. Empirical evidence suggests that increased funding contributes to improved performance and health outcomes, particularly in low‐ and middle‐income countries [38]. Conversely, reductions in government funding have been linked to declines in health system efficiency [39]. Jing et al. (2020) [40] highlight that disparities in the availability of health resources for persons with disabilities across regions of China are largely attributable to differences in regional economic development.

The combined model for all four types of disability revealed that an increase in the number of PWD within a health region does not necessarily increase the likelihood of CER implementation in its territory. This finding is consistent with previous research [41, 42]. However, when analyses are stratified by type of disability, significant associations are observed between the number of persons with specific disabilities and the presence of CERs targeting those conditions. These results suggest that other factors—such as the pre‐existing availability of rehabilitation services, advocacy from civil society, and political interests—may play a critical role in determining the territorial distribution of CERs.

It is imperative that health managers and policymakers recognize the territorial distribution of rehabilitation services as a critical determinant of both access and equity. PWDs require continuous and coordinated care; however, they frequently report transportation‐related challenges [1] and encounter significant barriers to accessing health services [43]. A more equitable spatial distribution of health infrastructure and services is essential to reducing these access gaps [44]. Individuals residing in regions with limited service provision and persistent access barriers tend to utilize healthcare services less frequently [45] and face greater difficulties in accessing specialized and high‐complexity care [36]. The current configuration of service distribution thus contributes to the perpetuation of regional disparities in healthcare access and health outcomes.

In addition to spatial disparities, various other barriers hinder persons with disabilities (PWDs) from accessing and maintaining continuous care. These include logistical factors—such as the distance to health facilities, inadequate or costly transportation—physical and communicational accessibility, attitudinal barriers, discrimination by health professionals, and the lack of technical knowledge and communication skills among service providers [46]. The availability of rehabilitation professionals is also unevenly distributed across the national territory, with a greater concentration in urban centres [47]. Rehabilitation plays a crucial role within health systems, contributing not only to individual health and well‐being but also to the broader economic development of communities [48]. Globally, it is estimated that approximately 2.4 billion people could benefit from rehabilitation services [1].

The presence of CERs in only 141 out of 348 health regions underscores the limited integration of rehabilitation services within comprehensive health care networks. Current national health policies lack clear guidance on the optimal spatial distribution of CERs. However, based on the strategic framework for organizing regional healthcare networks, it is essential that each Health Region includes at least one CER to ensure adequate service coverage. Evidence from health systems that adopt a network‐based care model indicates higher user satisfaction [49], improved integration between primary health care (PHC) and specialized services [50], more efficient use of resources and reduced costs [51], enhanced coordination between managers and service providers [52], and a decrease in emergency hospitalizations for individuals with chronic conditions [53].

Common challenges in the provision of care for PWD in low‐ and middle‐income countries can be broadly categorized into two domains: (I) governance and (II) health services. Governance‐related challenges include insufficient political commitment, limited political will, and weak forms of community organization that advocate for the implementation of inclusive policies. In the domain of health services, key barriers include a general lack of awareness about existing services, shortages in the health workforce, poor coordination among different sectors of the health system, and difficulties in standardizing data related to PWD. These challenges are compounded by the absence of a shared conceptual understanding of disability, impairment, and rehabilitation [3, 46].

The Brazilian regulatory framework is generally supportive of the right to health for PWD. However, the mere existence of an adequate policy does not automatically lead to successful outcomes, as its effectiveness is contingent upon factors such as the availability of resources, social support, the inclusion of diverse stakeholder groups in decision‐making, and the commitment of actors responsible for implementing the policy on a day‐to‐day basis [54]. Addressing these issues is crucial for the comprehensive implementation of a health services network for PWD.

The implementation of CERs is carried out through agreements between municipalities, Brazilian states, and the Ministry of Health. The municipalities are responsible for managing and allocating financial resources for services, while the Ministry of Health oversees the construction and establishment of these services. Consequently, one potential recommendation to reduce disparities in access to specialized rehabilitation services would be to prioritize regions with the greatest rehabilitation needs.

The limitations of this study include the use of data from the 2010 national census, which was delayed due to the Covid‐19 pandemic. As a result, the census, initially scheduled for 2020, has yet to be conducted, and updated data on the number of persons with disabilities in Brazil remains unavailable. Another limitation is the methodological choice to rely solely on secondary data; incorporating interviews with policymakers and managers responsible for implementing the health care networks would have enhanced the study's findings.

## 4 Conclusion

The Brazilian federal system, characterised by a tripartite division of legislative powers among the Union, States, and Municipalities, adds complexity to understanding the phenomenon under investigation. As this is a quantitative study, information regarding the political disputes surrounding the implementation of services has been excluded from the analysis. Consequently, the implementation of rehabilitation services in Brazil remains a multifaceted process that warrants further examination, particularly concerning the political conflicts that have emerged in the context of service delivery.

Standardized by the Brazilian federal government in 2012, the RCPD aims to create a collaborative network of integrated health services that addresses the longitudinal needs of PWD. However, the funding for this initiative is specifically allocated to CERs.

When considering the four types of disabilities individually, both the proportion of PWD and the transfer of financial resources enhance the likelihood of CER implementation. However, when analysing all disabilities collectively, the implementation of CERs does not correlate with health regions that have a higher population rate of PWD.

Prioritising the implementation of services based on the territorial distribution of needs is crucial to improving equitable access and quality of care. To achieve this, the use of accurate data to identify PWD is essential for the development of government programs and actions that address the population's specific needs.

Therefore, health care for PWD must be organised through interdependent and complementary actions and services, underpinned by effective communication between them. Comprehensive care will depend not only on specific technical interventions but also on interdisciplinary and intersectoral efforts.

## Ethics Statement

The authors have nothing to report.

## Conflicts of Interest

The authors declare no conflicts of interest.

## Data Availability

The data that support the findings of this study are available from the corresponding author, PHMS, upon reasonable request.
